# Morphological and Molecular Analysis of In Vitro Tubular Structures from Bovine Yolk Sac-Derived MSCs

**DOI:** 10.1155/2019/5073745

**Published:** 2019-03-06

**Authors:** Celina A. F. Mançanares, Vanessa Cristina de Oliveira, Lilian J. Oliveira, Maria A. Miglino, Flávio Vieira Meirelles, Carlos E. Ambrósio

**Affiliations:** ^1^Department of Veterinary Medicine, Faculty of Animal Science and Food Engineering, University of São Paulo, Pirassununga, São Paulo, Brazil; ^2^Department of Surgery, Faculty of Veterinary Medicine and Animal Science, University of Sao Paulo, São Paulo, Brazil

## Abstract

The yolk sac is an extraembryonic membrane, of saccular form, connected to the ventral region of the embryo. It is the main source of nutrition for the embryo during the period when the placenta is not fully formed. The aim of this study was to generate tubular structures using mesenchymal stem cells from the bovine yolk sac (bYS-MSCs) and determine if these structures can be a model for in vitro vasculogenesis. The evaluation of this tissue by histochemistry revealed a strong marking of collagen fibers and PAS technique negativity. In transmission electron microscopy, cytoplasmic organelles with large nuclei were observed. The vessel formation assay on a Matrigel substrate showed that the mesenchymal cells of the yolk sac without growth factors (VEGF) are capable of forming branches, sprouting cells, and tubular structures similar to capillary blood. These tubular structures were xenotransplanted subcutaneously into the mesentery of BALB/c/nude mice; after 45 days, vascularized tissue and extensions of blood vessels around the tubular structures could be observed. Real-time PCR (qPCR) demonstrated an expression of the VEGF gene in different gestational age groups. No difference in distribution or expression was detected among groups. Our results suggest that the spontaneous formation of tubules from the yolk sac can be an experimental model to elucidate initial organogenesis and the possible formation of blood capillaries from in vitro mesenchymal cells and possible route of organoid production.

## 1. Introduction

The yolk sac (YS) is a very important embryonic attachment in the study of embryonic development for cultivation and differentiation because it participates in important functions such as the formation of the primitive intestine and vascular system and the primordial germ cells that migrate to the developing gonads. Macroscopically, the bovine yolk sac is a transparent membrane composed of three regions: one central and two ventrally elongated projections, similar to the letter Y [1–3].

In mammalian embryos, hematopoiesis and vasculogenesis start in mesenchymal vascular islands of the yolk sac [4, 5], formed by large nucleated cells (hemangioblasts).

The mesoderm vascular islands are formed by hemangioblasts, cells with the ability to differentiate into hematopoietic and endothelial lineages, and they are renewed for the continuous production of all blood cells [5].

During embryogenesis, endothelial progenitor cells are involved in the initial process of primitive blood vessel formation (vasculogenesis). Endothelial progenitor cells concentrate in the bone marrow of adults, and they can be mobilized into the circulation by cytokine or angiogenic growth factor signals to promote the formation of the new blood vessels. For this reason, the use of autologous endothelial progenitors, mobilized in situ or transplanted, has become a major therapeutic approach for revascularization of ischemic diseases and endothelial injury [6].

Cell therapy with mesenchymal stem cells from the yolk sac is a promising therapeutic alternative, but this biological process is still unclear [7].

The yolk sac can be considered an excellent source for cellular plasticity due to its ability to produce mesenchymal stem cells [1] and hematopoietic stem cells [2]. Due to the potential of the YS to participate in these events during embryonic development and their unlimited self-renewal accompanied by high plasticity and cell differentiation, it is believed that this structure will be an invaluable model for cell therapy and for a better understanding of organogenesis and vasculogenesis.

Fetal membranes have recently been identified as an important stem cell source, which is ethically acceptable and easy to obtain, yet still causes minimal immunogenic problems. Considering the importance of this embryonic attachment and the capacity of mesenchymal cells to give rise to other tissues, we believe that it is possible to develop a new therapeutic approach such as transplantation, tissue regeneration, and in vitro vasculogenesis.

## 2. Materials and Methods

### 2.1. Laboratories, Material Collection, and Procedures

The protocols used for this research were approved by the research ethics committee (1.656743/2011) of the Faculty of Animal Science and Food Engineering, University of São Paulo, Brazil. The uterus was collected in the slaughterhouse via incision; the embryos were analysed by measuring the crown–rump (CR) length, following previously described methodology [3, 8]. As the yolk sac reduced in size during gestation, collected embryos were characterized into groups (*n* = 3) according to the CR measurement *-*25 to 34 days (crown–rump 0.3 to 1.4 cm) and separated into group I (25-29 days) and group II (30-34 days).

### 2.2. Isolation and Culture of bYS-MSCs

YS cells were cultured following the protocol of Mançanares et al. [1]. Cells were first washed in a solution of PBS-L (without calcium or magnesium) supplemented with antibiotics (5% penicillin-streptomycin; Invitrogen, Carlsbad, CA, USA) and then macerated enzymatically with 0.5% collagenase IV (Sigma-Aldrich) for 1 hour. The collagenase was inactivated with *α*-MEM culture medium (minimum essential medium, Gibco) supplemented with 10% fetal bovine serum (Gibco), BME amino acid solution 50x (Sigma-Aldrich), MEM nonessential amino acids 100x (Sigma-Aldrich), *β*-mercaptoethanol solution 100x (Gibco), and a 1% *v*/*v* antibiotic solution (penicillin G 10.000 units/mL and streptomycin 10.000*μ*g/mL). Cells were plated in 25 cm^2^ plates in a 37°C incubator with 5% CO_2_.

### 2.3. Formation of Tissues of Mesenchymal Origin

To promote the formation of tissues, bYS-MSCs (25 to 34 days of gestation) were cultured in *α*-MEM culture medium (minimum essential medium, Gibco) supplemented with 10% fetal bovine serum (Gibco), BME amino acid solution 50x (Sigma-Aldrich), MEM nonessential amino acids 100x (Sigma-Aldrich), *β*-mercaptoethanol solution 100x (Gibco), and a 1% *v*/*v* antibiotic solution (penicillin G 10.000 units/mL and streptomycin 10.000 *μ*g/mL) and were trypsinized (TrypLE Express; Gibco-BRL). When they reached 80% confluence, they were replaced. From passage 4, the cells started an aggregation process to spontaneously form a three-dimensional tubular structure. To evaluate the tubular structure, histology, transmission electron microscopy, and electronic microscopy techniques were performed.

### 2.4. Histochemistry and Light Microscopy

Tissues generated by bYS-MSCs at 20-34 days of gestation were used. Samples were fixed in 10% buffered formalin. After fixing, the material was dehydrated in a series of ethanol solutions with increasing concentration (70 to 100%), diaphanized in xylene, and followed by impregnation and inclusion in paraffin-like resin (Histosec®) (Tolosa et al., 2003) [9]. The materials were cut to 5 microns in a Leica RM 2145 microtome and placed on glass slides.

For light microscopy, the slides were heated at 37°C for 2 hours prior to staining. The tissues generated by mesenchymal cells were stained with hematoxylin and eosin (HE), toluidine blue, and picrosirius (total collagen fibers) (Tolosa et al., 2003) [9].

### 2.5. Transmission Electron Microscopy and Electronic Microscopy

The tissues generated by bYS-MSCs were fixed in Hagar and glutaraldehyde (2.5%, Sigma-Aldrich) for 24 hours. Then, the material was washed 3 times in 0.1 M phosphate buffer (pH 7.4) for 10 minutes. Subsequently, the material was postfixed in osmium tetroxide for 2 hours and again washed 3 times in 0.1 M phosphate buffer (pH 7.4) for 10 minutes. Dehydration was performed in batteries of 15 minutes with increasing concentrations of alcohol. The critical point obtained by the apparatus (CPD 020, Balzers Union) where all the ethanol was removed completely dried the material for plating with gold. As a last step of processing, the pieces were fixed in “stubs” of aluminum with carbon glue, maintained in an oven for 12 hours for drying, and subjected to a gold bath (Emitech, 550 K). The analyses and electron micrographs were made by a scanning electron microscope (LEO, 435 VP).

### 2.6. Rudimentary Formation Assay on Matrigel Vessels In Vitro

To investigate the potential formation of yolk sac vessels from stem cells, an in vitro test was performed using the BD Matrigel™ substrate (BD Biosciences, USA). As a positive control, bovine umbilical vein endothelial cells (BUVECs) cultured in vitro under the same conditions as those of bYS-MSCs. The Matrigel (BD Biosciences) was prepared according to the manufacturer's instructions.

The bYS-MSCs were plated at a concentration of 1 × 10^5^ cells in EGM-2 medium (Gibco-BRL, USA) with 10% FCS on the Matrigel substrate (Covas et al., 2008). Samples were viewed under phase-contrast microscopy (Olympus IX71), and pictures were recorded using a digital camera (Olympus U-TV0.5XC-3) at 0 hour, 10 hours, 24 hours, 5 days, 10 days, and 15 days. The area was quantified using the ImageJ software (NIH Image-BioLab). We did the comparative analysis of the umbilical cord and bYS-MSCs.

### 2.7. Polymerase Chain Reaction

Total RNA (from purified samples) was isolated using a mini kit (QIAGEN) according to the manufacturer's instructions, and the RNA was quantified by UV spectrophotometry at 260 nm; triplicates were used for each group. The cDNA High-Capacity Reverse Transcription kit (Applied Biosystems, Foster City, CA, USA) was used for cDNA synthesis using random primers. Gene expression was assessed by quantitative PCR (StepOne Real-Time PCR Systems; Life Technologies, Carlsbad, CA, USA). The reactions were performed using a commercial assay system (SYBR® Green PCR Master Mix; Life Technologies) with VEGF as the target reporter gene and GAPDH as the housekeeping gene. The sequences of the primers used are shown in Table 1. The reaction conditions consisted of 40 cycles with an annealing temperature of 60°C. VEGF was evaluated by normalizing the signals against GAPDH signals using the 2^-ΔΔCt^ method.

### 2.8. Xenotransplantation

For the xenotransplantation protocol, the tissue generated by bYS-MSCs was fixed in the mesentery and sacral region in 3 animals (BALB/c/nude). For this procedure and in accordance with ethical principles, the animals were anesthetized with ketamine (6.7 mg/mL) and xylazine (1.3 mg/mL) given their 0.15 g mL/body weight. The tissue generated by bYS-MSCs was added close to the mesentery with a large caliber vessel for guaranteed nutrition of this tissue. Animals were monitored daily and maintained in intensive care for 45 days.

After 45 days, the animals were euthanized following ethical principles. The animals were fixed by perfusion processes, injecting aqueous 10% formaldehyde via the external jugular vein, and immersed in the same fixative, remaining submerged for at least 48 hours. Later, the animal was dissected and regions to be processed for microscopy were cut, placed in cassettes, and placed in 70% alcohol. The regions were photographed by a Sony Mavica 3.2 MP digital camera.

## 3. Results

### 3.1. Isolation, Culture, and Formation of Tissues of Mesenchymal Origin

The hematopoietic and mesenchymal cells from bovine yolk sac were previously characterized by Mançanares et al. and Oliveira et al. [1, 2] from our group.

**Figure 1:**
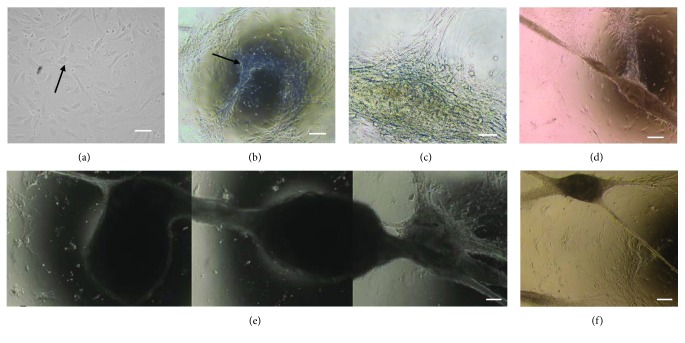
Photomicrographs of the tubular structure formed from bYS-MSCs. Fibroblast-like morphology that adhered to the plastic culture surface (arrow) (a); adherent cells began to organize into a network with a cobweb appearance (b–d); tubular structures (e, f). Scale bar: 50 *μ*m.

The bYS-MSCs were adherent with fibroblast-like morphology (Figure 1(a)). After 4 passages, the cells started a matted organization (Figures 1(b)–1(d)) and began to form tubular structures, similar to blood vessels, after 15 days in culture (Figures 1(e) and 1(f)).

### 3.2. Rudimentary Vessel Formation Assay on Matrigel

The vessel formation assay on Matrigel showed that the mesenchymal cells of the yolk sac without growth factors (VEGF) are capable of forming branches, sprouting cells, and similar structures of capillaries.

**Figure 2:**
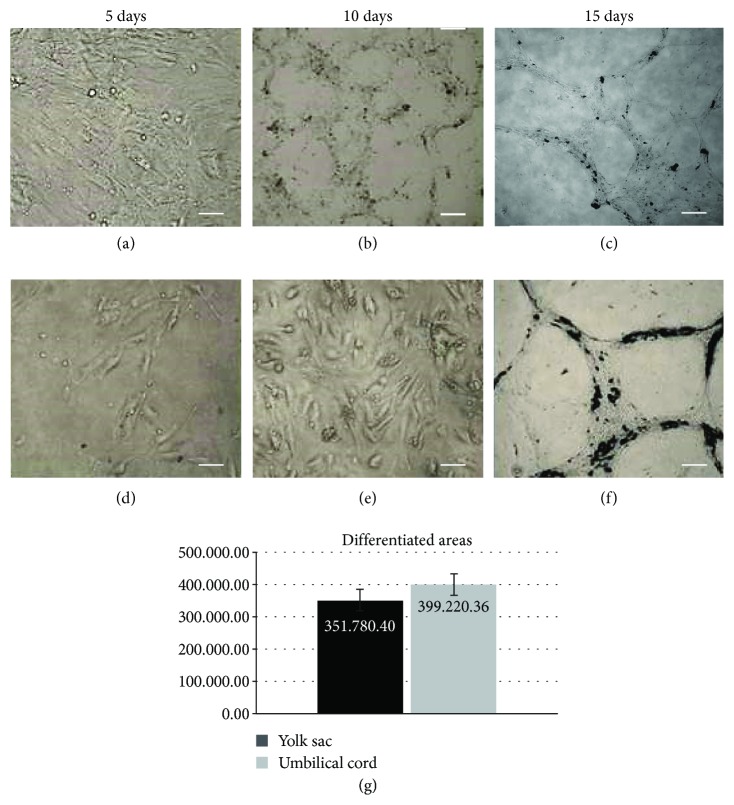
Capillary tubule formation assay of the bYS-MSCs and umbilical cord (CB) differentiated in vitro on Matrigel. Control of umbilical cord (a–c) with 5, 10, and 15 days of culture; bYS-MSCs (d–f) with 5, 10, and 15 days of culture. Scale bar: 50 *μ*m. In (g), note the comparative analysis of the differentiated area of the umbilical cord and bYS-MSCs.

For this substrate assay, the bovine umbilical cord was used as a positive control. After 15 days, the bYS-MSCs started to organize into matted and capillary-like structures (Figure 2). Umbilical cord cells and yolk sac showed similar results regarding morphology and differentiation. The total and differentiated areas of the umbilical cord and yolk sac were measured by the ImageJ software, not showing significant difference (Figure 2(g)).

### 3.3. Microscopic Evaluation

**Figure 3:**
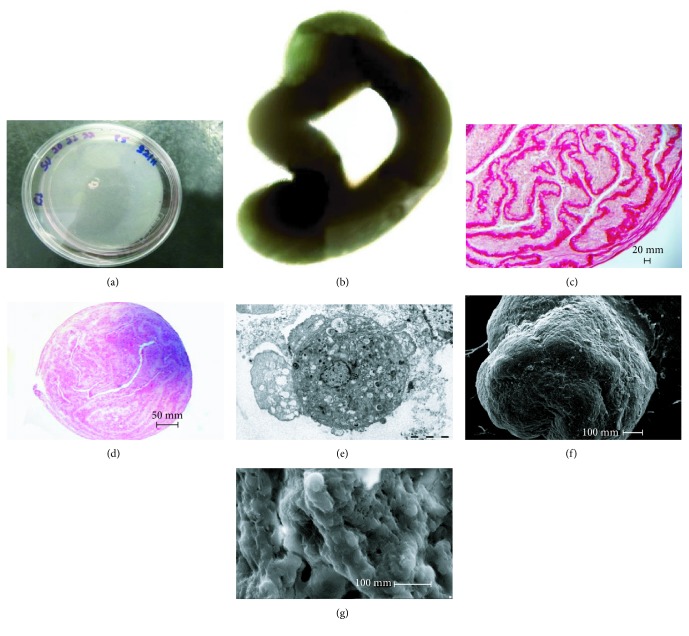
Photomicrographs of tubular structures formed from bYS-MSCs; electronic microscopy and transmission electronic microscopy. (a) Tubular structures formed on the plate; (b) tubular structure; (c) picrosirius staining, note bundles of strongly acidophilus collagen fibers; (d) HE staining of well-defined lumen; (e) transmission electronic microscopy analysis, cells grouped with broad and granular cytoplasm, the presence of cytoplasmic organelles and a large core; (f) and (g) microscopy analysis, note the irregular surface with uneven elevation, regions with spongy and/or scaly appearance, surrounded by a membrane of apparent connective tissue and tissue similar to blood vessels.

The tubular structure was observed only in the embryos of the 24-35-day group and showed irregular morphology, emphasizing its migratory and proliferative capacity (Figure 3(b)).

The tubular structures generated by the bYS-MSCs (Figures 3(a) and 3(b)) were processed and stained with HE (hematoxylin/eosin) and picrosirius. In histochemical reactions, staining with picrosirius clearly showed heavily stained collagen fibers arranged side by side (Figure 3(c)). Among the collagen bundles, fibroblasts and fibrocytes with their characteristically elongated and flat nuclei were noted. PAS staining (periodic acid-Schiff) was negative for this reaction. In HE analysis, it was possible to note the well-defined lumen (Figure 3(d)).

In transmission electronic microscopy evaluation, the tubules revealed a cell population that was clustered with a large and granular cytoplasm, an evident nucleus, and cytoplasmic organelles; likewise, cells with apoptotic characteristics were visualized (Figure 3(e)).

The microscopic evaluation showed a surface with elevations and some regions with spongy features surrounded by connective tissue. In a transverse section, the cells were clearly similar to the blood vessels (Figures 3(f) and 3(g)).

### 3.4. Xenotransplantation

At 45 days after xenotransplantation, the animals were fixed in 10% formalin. After an incision in the median mesogastric region, the abdominal organs were exposed to evaluation by a veterinary pathologist.

Under the stereoscopic microscope, this appears as a transparent tissue without definition that is involved in the borders of structures such as blood vessels. The remainder of the thread appears surrounded by vascularized tissue of mesenteric origin or tissue generated by bYS-MSCs. We did not find blood vessel extensions of the xenotransplantation structure, as expected. Perhaps these structures did not continue their development due to the absence of growth factors that are important modulators of cell growth and differentiation and are crucial to the stability of capillaries formed in vitro.

**Figure 4:**
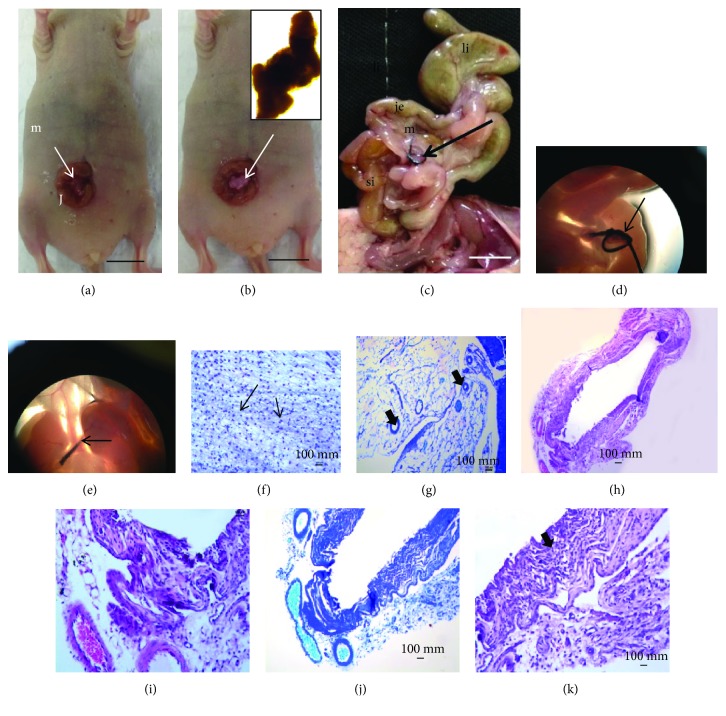
Photographs and photomicrographs of xenotransplant tissue generated by bYS-MSCs in immunosuppressed mice (nude). (a) Tissue prior to xenotransplantation (40x); (b) abdominal incision with tissue (arrow); xenotransplanted tissue (b); (c) gastrointestinal organ “ex situ,” observe the lack of growth of the structures in this region. Large intestine (li), jejunum (J), mesentery (m), and small intestine (si); (d, e) the mesentery (ms) and the suture in the tissue generated by the MSC center node (arrows); (f–k) HE staining and toluidine blue, note the collagen fibers (arrow) and elastic (thin arrow).

The mesentery was presented as a thin semitransparent fan-shaped membrane supporting the jejunum and the ileum and involving a large amount of blood vessels and nerves. The small intestine, the large intestine, and the remainder of the mesentery were carefully examined, and nothing was observed. When stained by HE and toluidine blue, collagen and elastic fibers were observed (Figure 4).

**Figure 5:**
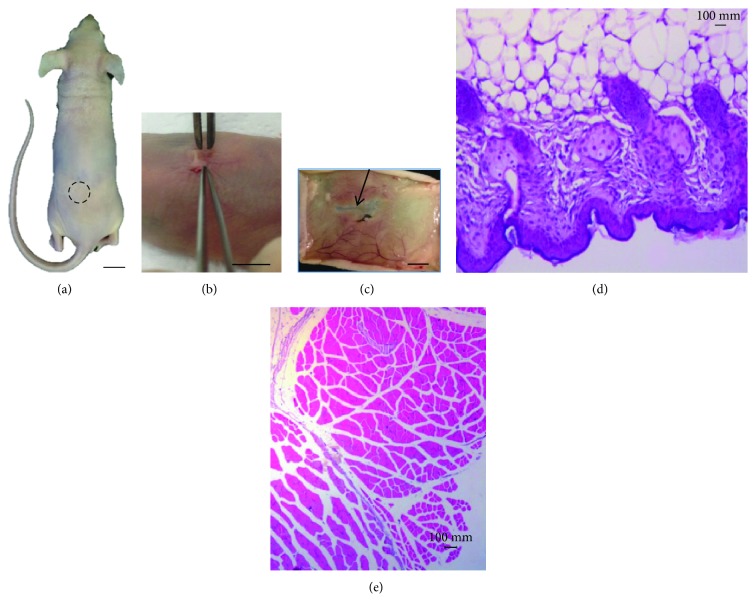
Photography of structures generated by bYS-MSCs after 45 days of xenotransplantation into immunosuppressed mice (nude). (a, d) The xenotransplantation was performed and the structures generated by bYS-MSCs (circle); (b) implementation of the tissue; (c) skin showing vascularized areas (thin arrow); (e) characteristics of the skin and muscles without morphological alterations (HE).

The skin in the subcutaneous region did not show the transfixed tissue in any of the animals studied at a macroscopic level, only a scar at the site of surgical incision where the tissue generated by bYS-MSCs was inserted. When stained with HE, the characteristics of the skin and muscles were without morphological alterations (Figure 5).

### 3.5. Polymerase Chain Reaction and Statistical Analysis

**Figure 6:**
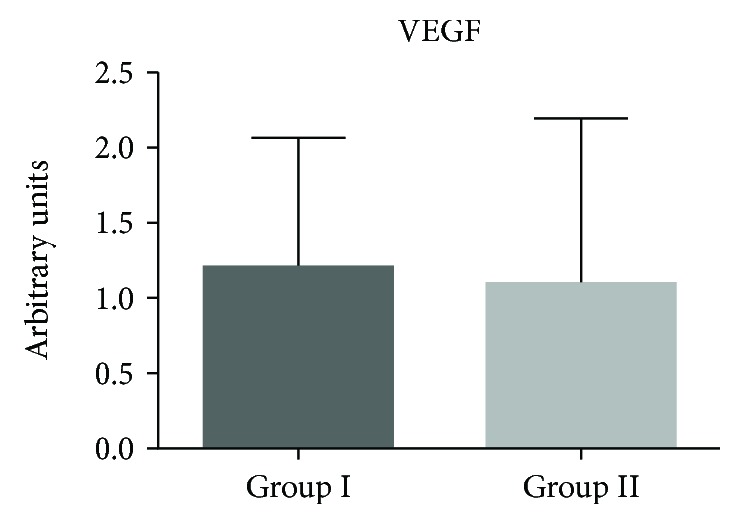
Expression of the VEGF gene using qPCR on bYS-MSCs from group I (25 to 29 days) and group II (30 to 34 days).

Structures formed at different embryonic ages were tested and divided into 2 groups: group I (25-29 days) and group II (30-34 days). VEGF was chosen for a positive control; this gene is highly specific for vascular endothelial cells. We observed an expression of this gene in both groups. PCR data were tested for the normality of their residues, using the Shapiro–Wilk test. Samples that did not meet the assumptions of normality were transformed into natural logarithms. The results were analysed using analysis of variance (PROC ANOVA) and means were compared via Tukey's test, using SAS software v. 9.2 (SAS Institute, Cary, NC, USA). A level of significance of 5% (*p* < 0.05) was considered for all experiments. Statistical differences were not observed in the expression levels between groups (Figure 6).

## 4. Discussion

During development in mammals, the yolk sac is the first source of stem cells and has niches of hematopoietic and mesenchymal cells [1, 2, 5, 10].

It is known that extended cultivation of MSCs may lead to detachment of cells as sheets that may form thread-like constructs. This is due to the contractile forces exerted by cells and differential adhesion to culture dishes. Many studies have shown that the MSCs can form 3D aggregates in vitro under mechanical forces. This mechanism of cellular aggregation is beginning to be investigated [11].

De Francesco et al. [12] showed that human CD34^+^/CD90^+^ cells of human adipose tissue are capable of forming sphere clusters (cellular aggregates) in culture, producing high levels of VEGF and forming capillaries. In our findings, the tubular tridimensional structure was formed in vitro without any growth factors. We used the protocol established by Mançanares et al. [1] to grow the bYS-MSCs. Based on our findings, we believe that the YSMSC can be a model of spontaneous vasculogenesis in vitro.

Li et al. [13] and Senegaglia et al. [14] also observed the same structures in their studies with the yolk sac and umbilical cord. However, the time of formation of these structures was different from our studies.

Li et al. [13] showed that the yolk sac cells form a complete capillary network between 12 and 24 hours. Senegaglia et al. [14] noted that after 24 hours of in vitro cultivation, the cells from the umbilical cord formed capillary tubules on Matrigel substrate.

Functional testing through tubular formation without Matrigel showed that the yolk sac cells in vitro had the formation of structures similar to capillaries, providing additional evidence that the cells proliferated and gave rise to cells similar to endothelial cells, in agreement with the findings of Senegaglia et al. [14] who used cells from the human umbilical cord.

Putnam and Mooney [15] isolated PECAM1-positive cells and subsequently seeded them on biodegradable polymer scaffold, and these highly porous structures were implanted subcutaneously in rats. In our studies, no cell type was separated or isolated, and we had similar results here.

Terranova et al. [16] describe in their study that the capacity of cell migration is essential for forming new vessels and capillaries and is a characteristic of endothelial cells that are capable of organization that results in the formation of three-dimensional tubular structures in vitro. In our studies, we observed that yolk sac cells have the potential to form structures similar to blood vessels with or without substrate (Matrigel). We observed that the mesenchymal cells of the yolk sac have cell migration potential, which is essential for forming new vessels and capillaries and is a characteristic of endothelial cells. These cells are capable of organization that results in the formation of three-dimensional tubular structures in vitro.

Some studies have shown that various substrates can induce the formation of endothelial cells: Matrigel alone [13], Matrigel with growth factors [16], collagen gel supplemented with laminin tubes [17], fibroblasts of fetal mice (MEF) and Matrigel [18], Matrigel and bFGF [19], and others. However, in our studies without the growth factor or substrate, structures such as blood vessels also formed, although the time to form those vessels increased.

In the xenotransplantation analysis, blood vessel extensions were not found; these structures likely stopped their development due to missing growth factors that are essential for modulation and differentiation. Chemotactic behavior is a property of a variety of cell types involved in biological processes and the development of organs. The endothelial growth factor (ECGF) has been implicated in neovascularization since 1985 [16]. The expansion phase in the presence of growth factors is crucial for the stability of in vitro capillaries [14].

The chemiomatic behavior is a property of a variety of cell types involved in biological processes and organ development. During embryogenesis, endothelial progenitor cells participate in the early processes of primitive blood vessel formation, where the endothelial progenitor cells reside in the bone marrow of adults and can be mobilized in the circulation by means of cytokine signals or growth factors angiogenic and promoting the formation of a new blood vessel. For this reason, autologous endothelial progenitors, mobilized in situ or transplanted, have become one of the main targets of therapeutic approaches for revascularization in ischemic diseases and endothelial injury [20].

The transplantation of yolk sac cells from mice into intrauterine halogenated fetuses and into xenogeneic newborns did not induce graft rejection reactions, which indicates that YS cells may be useful as universal donors for various species of mammals, including humans [21].

Any morphological changes were found in the intestine, near the area fixed with the bYS-MSC structure. All tissues were preserved, similar to the results found in the literature [22].

The microscopic analysis of the fixed structure in the mesentery showed that this structure presented a tubular formation with a lumen inside, surrounded by connective tissue in layers. The anatomic-pathological study of the skin after xenotransplantation was carefully assessed, and it was found that there was no formation of the blood vessels from this structure. The skin showed a keratinized stratified squamous epithelium with normal characteristics as well as the cornea, lucid, granulosum, spinosum, and basal and papillary layers [22].

The muscles of the lower back close to the application of the tubular structures showed microscopically normal morphology. The transverse and longitudinal skeletal muscle fibers had organized striations, with the nuclei located in the periphery without any alterations in this tissue [23].

There was also no macroscopic and microscopic evidence of inflammatory process in the skin and mesentery in the animals studied, and these observations are responsible for the low therapeutic efficacy after transplantation of cells or grafts in humans [24].

Fratini et al. [25] in their findings after the transduction of VEGF in canine embryonic yolk sac cells showed that is possible to characterize them as endothelial progenitor.

Melero-Martin et al. [26] showed that endothelial progenitor cells from umbilical cord blood and peripheral blood from the saphenous vein of adult humans, when cultured in endothelial basal medium (EBM-2) supplemented with SingleQuots, form vascular network organs and artificial tissues when implanted in the subcutaneous tissue of mice.

The vascularization of tissues is a major challenge for tissue engineering, and new studies should be conducted to isolate endothelial progenitor cells derived from the yolk sac.

Additionally, adding mesenchymal cell growth factors to the culture medium to establish their proliferative and vasculogenic activity is necessary to create vascular networks in vivo.

The more undifferentiated a cell is, the greater its potential for use in multiple applications. Thus, the presence of markers is important for confirmation and differentiation of various cell types [27].

Self-renewal and pluripotency of embryonic stem cells are regulated by a complex network of events, consisting of cellular factors in the microenvironment, a chain of transitional factors, and probably signal transduction pathways [13]. Thus, to evaluate the performance of VEGF (vascular endothelial growth factor) via Flt-1 and KDR receptors on embryonic stem cells, the bovine yolk sac can help elucidate the process of differentiation of these cells and enable new prospects for cell therapy.

Wang et al. [28] also investigated the KDR and VEGF in the yolk sac and liver of a human embryo. They observed the expression of KDR/VEGF and suggested the presence of hemangioblast in the yolk sac and liver, showing that this presence can be related to survival, proliferation, migration, and differentiation of hemangioblasts.

For analysis of gene expression, we used two gestational age groups (groups I and II). These analyses found VEGF to be expressed in both groups, and the expression was approximately equal in group I and group II. The results agree with the findings of Marinovic [29] wherein these genes are expressed within 45 days of gestation in the yolk sac of bovine embryos. Its expression shows that the mammalian yolk sac of the embryos contains hemangioblasts, which are progenitors able to differentiate into hematopoietic cells and vascular endothelial cells through hematopoiesis and vasculogenesis processes [30].

VEGF is a highly specific gene for vascular endothelial cells. It induces endothelial cell proliferation, promotes cell migration, inhibits apoptosis and angiogenesis, and induces the regulation of vascular formation [31].

Regarding angiogenic factors, the gene expression of VEGF present in the yolk sac was studied showing that it continues to perform its function, a fact described by [30, 32] who identified the VEGF system in the placenta of various species, including bovine. In our results, its receptors are found during pregnancy in endothelial, endometrial, and trophoblast cells.

The vascularization of tissues is a major challenge for tissue engineering; new studies should be conducted both for the isolation of only the progenitor cells derived from the yolk sac and for the addition of mesenchymal cell growth factors to the culture medium to determine if they have the proliferative activity and vasculogenic necessary to create vascular networks in vivo.

## 5. Conclusion

We conclude that the tissue generated by the mesenchymal cells of the yolk sac formed blood vessel-like structures without growth factors and was positive for VEGF expression. VEGF gene expression was positive for bovine embryo cells cultured in groups I and II, showing that the yolk sac may be of importance in vascular development.

This study was a first step towards understanding the importance of the yolk sac from the morphological point of view as a molecular and experimental model for the development of new lines of research related to stem cells and cell therapy, thereby providing insight into the biology of hematopoiesis linked to the formation of organs in the embryo. The construction of stable blood vessels can be a fundamental challenge for tissue engineering in regenerative medicine. These results can contribute to the development of new techniques for cultivation of yolk sac tissue for the formation of vasculogenesis and angiogenesis and may provide the proliferative and vasculogenic activity necessary to create vascular networks in vivo.

## Figures and Tables

**Table 1:** Primers designed for qPCR [^a^forward = sense (5′) primer, ^b^reverse = antisense (3′) primer].

Gene name	Accession	Primer	Sequence (5′-3′)
*Bos taurus* vascular endothelial growth factor A (VEGF)	NM_174216.1	Forward^a^	GCCCACTGAGGAGTTCAACAT
		Reverse^b^	CTGGCTTTGGTGAGGTTTGATC

## Data Availability

No data were used to support this study.
